# Chronic obstructive pulmonary disease and asthma: mesenchymal stem cells and their extracellular vesicles as potential therapeutic tools

**DOI:** 10.1186/s13287-022-02938-5

**Published:** 2022-06-20

**Authors:** Hossein Abbaszadeh, Farzaneh Ghorbani, Sanaz Abbaspour-Aghdam, Amin Kamrani, Hamed Valizadeh, Mehdi Nadiri, Armin Sadeghi, Karim Shamsasenjan, Farhad Jadidi-Niaragh, Leila Roshangar, Majid Ahmadi

**Affiliations:** 1 Stem Cell Research Center, Tabriz University of Medical Sciences, Tabriz, Iran; 2 Tuberculosis and Lung Disease Research Center of Tabriz University of Medical Sciences, Tabriz, Iran; 3 Immunology Research Center, Tabriz University of Medical Sciences, Tabriz, Iran

**Keywords:** Lung diseases, COPD, Emphysema, Asthma, Exosomes, Microvesicles, Regeneration, Mesenchymal stromal cells

## Abstract

Chronic lung diseases, such as chronic obstructive pulmonary disease (COPD) and asthma, are one of the most frequent causes of morbidity and mortality in the global. COPD is characterized by progressive loss of lung function through inflammation, apoptosis, and oxidative stress caused by chronic exposure to harmful environmental pollutants. Airway inflammation and epithelial remodeling are also two main characteristics of asthma. In spite of extensive efforts from researchers, there is still a great need for novel therapeutic approaches for treatment of these conditions. Accumulating evidence suggests the potential role of mesenchymal stem cells (MSCs) in treatment of many lung injuries due to their beneficial features including immunomodulation and tissue regeneration. Besides, the therapeutic advantages of MSCs are chiefly related to their paracrine functions such as releasing extracellular vesicles (EVs). EVs comprising exosomes and microvesicles are heterogeneous bilayer membrane structures loaded with various lipids, nucleic acids and proteins. Due to their lower immunogenicity, tumorigenicity, and easier management, EVs have appeared as favorable alternatives to stem cell therapies. Therefore, in this review, we provided an overview on the current understanding of the importance of MSCs and MSC-derived EVs from different sources reported in preclinical and clinical COPD and asthmatic models.

## Introduction

Chronic respiratory diseases, such as asthma and chronic obstructive pulmonary disease (COPD), have represented an extremely high social burden. COPD is a common and progressive lung condition, associated with irreversible airflow obstruction and airway inflammation which have adverse effects on the patient’s breathing and restrict lung function, measured by total lung capacity and forced expiratory volume (FEV) [1–4]. Furthermore, chronic cough, wheezing, dyspnea, as well as sputum and phlegm are the other common symptoms for COPD [4, 5]. According to the World Health Organization (WHO) prediction, COPD will become the third leading cause of morbidity and mortality across the globe in 2030 [6] and represents a considerable economic burden on the individual and society [7]. Long-term exposure to noxious particles or gases, particularly cigarette smoke (CS), is the major risk factor for developing COPD [8, 9]. CS exposure induces increased number of neutrophils, lymphocytes, and macrophages and cause inflammation in the small airway and lungs through releasing different pro-inflammatory mediators [8, 10]. Asthma is also a heterogeneous disorder which regularly indicates development of airway obstruction [11, 12]. It is characterized by airway inflammation, bronchospasm and amplified mucus production inside the airways [13, 14].

Current available treatment methods for COPD and asthma such as anti-inflammatory drugs, corticosteroids, long-acting muscarinic antagonists and β2-adrenoceptor agonists may help slow the progression of these disorders but cannot reverse the lung damage or improve the quality of life in these patients [1, 15–17]. Therefore, more research efforts are required to better understand the molecular mechanism of COPD and asthma pathogeneses and development of new therapeutic and diagnostic approaches.

Increasing data have specified that mesenchymal stem cells (MSCs) are great means for cell-based therapy and regenerative medicine because of their multipotent differentiation and self‐renewal abilities and immunoregulatory properties, as well as long‐term ex vivo proliferation and paracrine effects [18–21]. MSCs have been widely studied in respiratory diseases including COPD, asthma, and idiopathic pulmonary fibrosis, and are powerful candidates for regeneration of lung damages [4, 16, 22–24]. Additionally, it has been proven that the beneficial effects of MSCs are mostly associated with their paracrine factors, especially extracellular vesicles (EVs) [25, 26]. EVs including exosomes, microvesicles (MVs), and microparticles (MPs), and their miRNAs content, have been widely reported as potential therapeutic means for a variety of lung diseases and they also serve as signaling molecules to mediate cell–cell communications, particularly between epithelial cells and lung microenvironment [27–29]. As compared with their parental MSCs, these vesicles represent a better safety profile and can be stored without missing function [30].

**Fig. 1:**
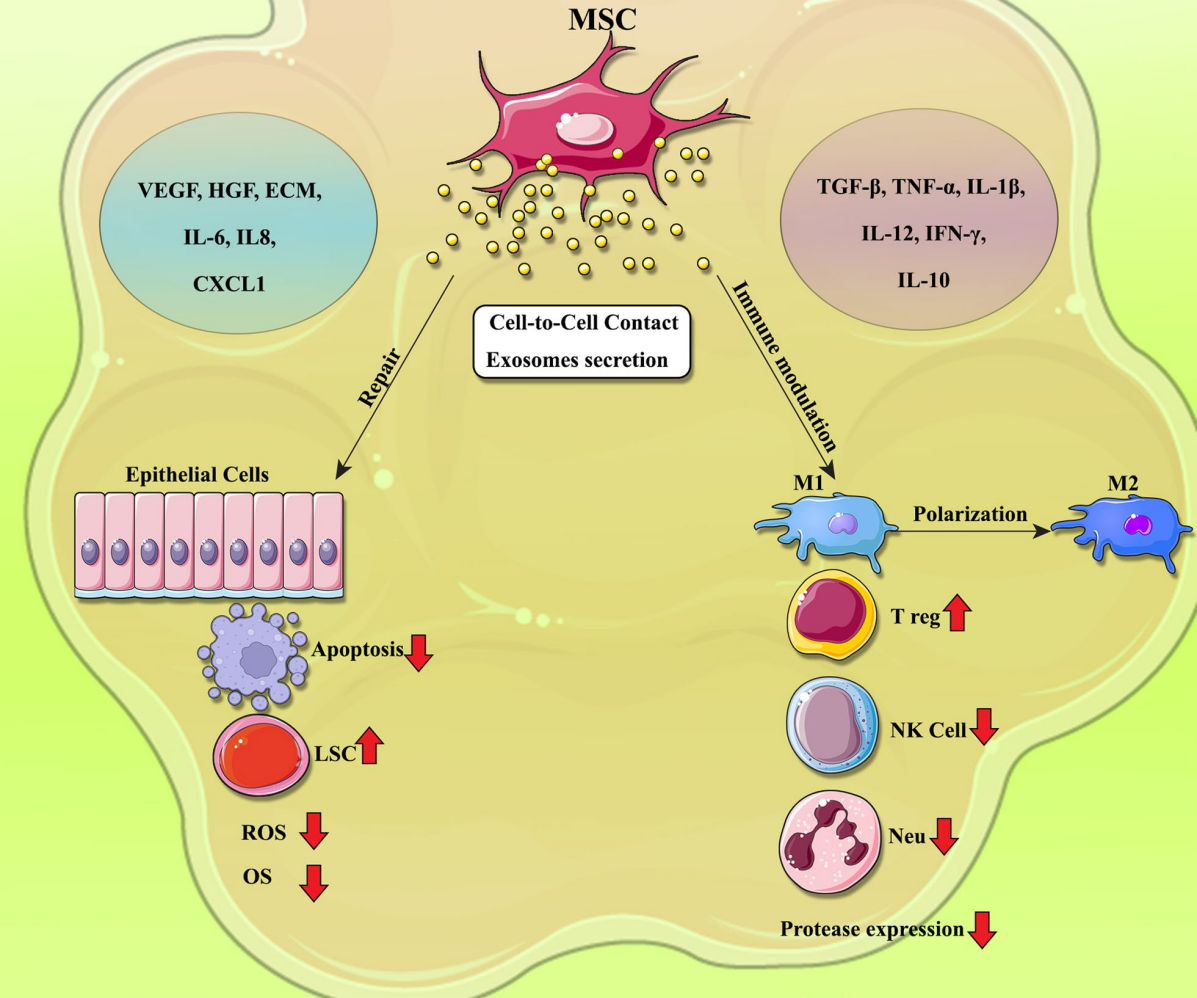
Mechanisms underlying the modulation of inflammation and lung tissue repair by Mesenchymal stem cells (MSCs) in COPD

**Fig. 2:**
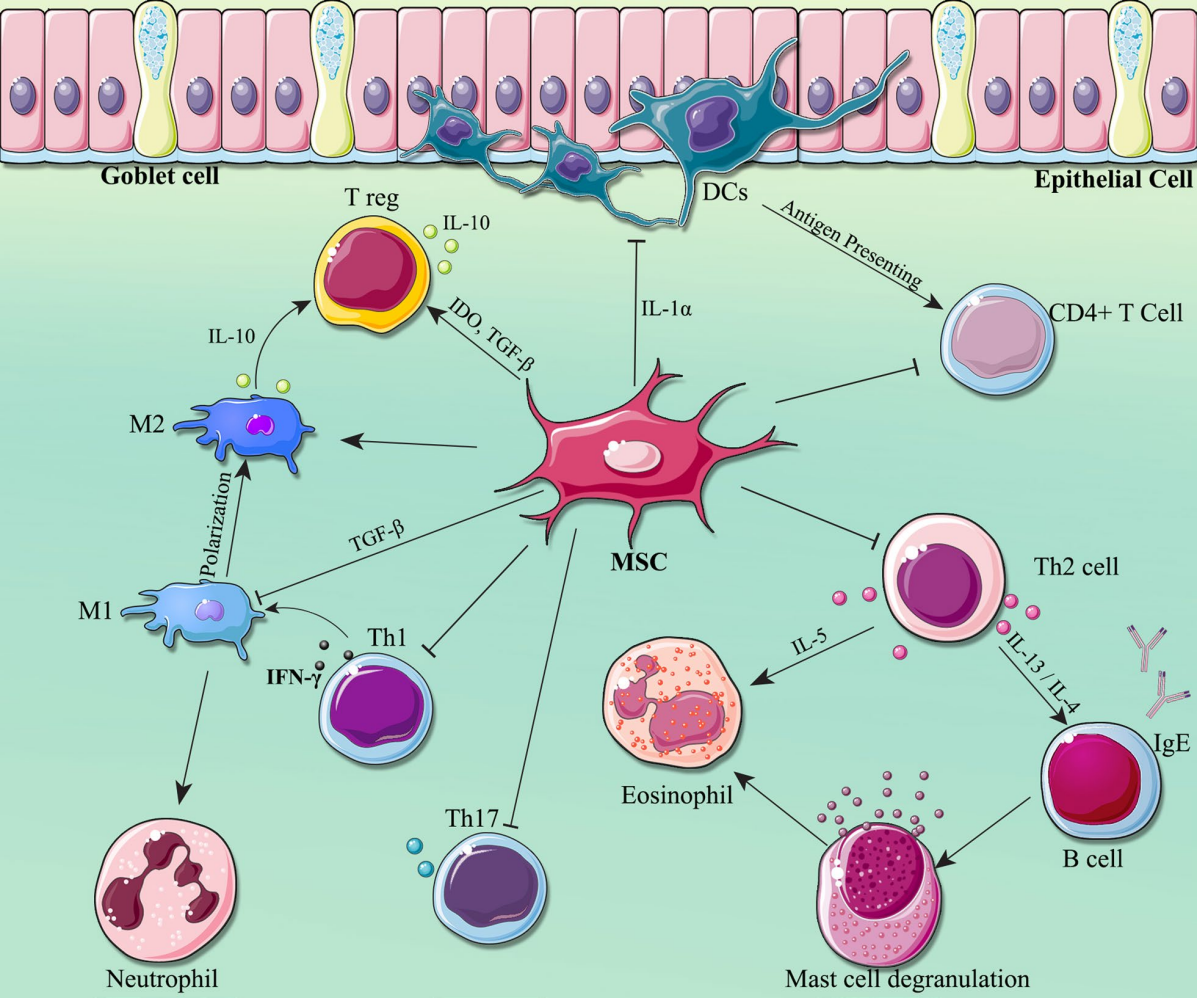
Immunomodulatory effects of Mesenchymal stem cells (MSCs) on immune cells in asthma condition

Accordingly, in this review, we will summarize current knowledge about the role of MSCs and their EVs, principally exosomes, in treatment of COPD and asthma disorders (Fig. 1 and Fig. 2).

## MSCs and their EVs

MSCs are multipotent adult stem cells which have become extensively studied during the past 30 years in both preclinical and clinical trials for numerus disorders [31, 32]. They can differentiate into multiple lineages, including osteocytes, adipocytes and chondrocytes and can be found approximately in all postnatal organs such as bone marrow (BM), adipose tissue (AD), human-induced pluripotent stem cells (hi-PSC), Wharton’s Jelly (WJ), human cord blood (hUC), umbilical cord (UCB), and human amniotic membrane (hAM) as well as human placental (hP) and dental follicle [33–35]. In general, these MSCs express surface markers, such as CD105, CD90, and CD73, but not CD34, CD45, CD19, CD11b, CD79α, and CD14 [36–38]. MSCs pose broad anti-inflammatory, immunoregulatory and regenerative features [39–42]. It has been elucidated that EVs secreted from MSCs have key role in mediating biological processes, for example blood coagulation, stem cell differentiation, immunomodulation, and regeneration as well as angiogenesis and etc. [43–45]. EVs are nanosized vesicles that have two main subtypes including exosomes which are sized in 30 to 150 nm in diameter generated from secretion of microvesicular bodies into extracellular space. MVs are other main types of EVs that are sized between 100 to 1000 nm and formed by direct budding from the plasma membrane [32, 46, 47].

## Various MSCs in treatment of COPD and asthma

As demonstrated in Tables 1 and 2, accumulating studies have recently examined the potential contribution of MSCs derived from different sources including BM, hi-PSCs, WJ, hCB, UC, AD and hAM as well as hP and dental follicle in regeneration and treatment of COPD and asthma, which are discussed in the next parts.

**Table 1:** Therapeutic application of various mesenchymal stem cells and their extracellular vesicles in preclinical COPD and asthma models

Injury	Study type	type of MSCs	Infusion method	Dose of injection	Outcome	Reference
COPD	NCI-H292 airway epithelial cells	TNF-α and IL-1β-activated BM-MSCs	–	–	Increased airway epithelial wound healing via activation of the epidermal growth factor receptor	[48]
COPD	Mice model	BM-MSCs	Intravenous	4 × 10^6^ cells/mL	Relieved lung injury through promoting proliferation of endogenous lung stem cells	[49]
COPD	Rat model	BM-MSCs	Intratracheal	6 × 10^6^ cells/mL	Protect cigarette smoke-damaged lung and pulmonary function partly via VEGF–VEGF receptors	[50]
COPD	Mice model	BM-MSCs	Intravenous	4 × 10^6^ cells/mL	Ameliorate lung injury through anti-inflammatory and anti-bacterial effect	[51]
COPD	Rat model	BM-MSCs	Intratracheal	6 × 10^6^ cells/mL	Alleviated airway inflammation and emphysema through down-regulation of cyclooxygenase-2 via p38 and ERK MAPK pathways	[52]
COPD	Mice model	BM-MSCs	Intravenous	5 × 10^5^ cells/mouse	Exerted HGF dependent cytoprotective effects	[53]
COPD	Rat model	BM-MSCs	Intravenous	2 × 10^6^ cells/rat	Inhibited the progression of emphysema by differentiating into endotheliocytes and suppressing the apoptosis of endotheliocytes and oxidative stress	[54]
COPD	Mice model	HSP-VEGFA-BM-MSCs	Intravenous	–	Alleviated elastase-induced emphysema	[55]
Asthma	Mice model	BM-MSCs	Intravenous	10^6^ cells/mouse	Simvastatin and BM-MSCs combination therapy affects serum IgE as well as lung IL-13 and TGFβ levels more than BM-MSCs and simvastatin therapy alone	[63]
Asthma	Mice model	BM-MSCs	Intravenous	2.5 × 10^5^ cells	Controlled inflammation, immune-inflammatory factors and mitochondrial related genes, and prevent asthma immune-pathology	[64]
			Bronchial	2.5 × 10^5^ cells		
Asthma	Mice model	BM-MSCs	Intratracheal	10^5^ cells/mouse	Released different mediators and differentially affected airway and lung parenchyma	[65]
		AD-MSCs				
		Lung-MSCs				
Asthma	Rat model	BM-MSCs	Intratracheal	2 × 10^6^ cells/rat	CM and especially MSCs ameliorated pathological changes via intratracheal route presumably by targeting ICAM-1 and VCAM-1 in lung tissues	[66]
Asthma	Mice model	BM-MSCs	Intraperitoneal	10^6^ and 2 × 10^6^ cells	Ameliorated to the airway remodeling and airway inflammation both in the upper and lower airways via the inhibition of Th2 immune response in the murine model of AR	[67]
Asthma	Rat model	BM-MSCs	Intravenous	–	Affected on Th1/Th2 drift, and the Notch1/Jagged1 pathway and may participate in the homing of the BM-MSCs	[68]
Asthma	Mice model	BM-MSCs	Intravenous	2 × 10^6^ cells/mouse	Significantly reduced total cells and eosinophilia and serum OVA-specific IgE concentration and inhibited expressions of Th2 and Th17 cytokines and elevated levels of Treg cytokines	[69]
Asthma	Mice model	BM-MSCs	–	–	Alleviated asthma by inducing polarization of alveolar macrophages	[70]
Asthma	Mice model	BM-MSCs	retro-orbital	10^6^ cells/mouse	Participated in improved outcomes of remodeling by reversing excess collagen deposition and changing hyaluronan levels	[71]
COPD	Mice model	ASMCs-treated iPSC-MSCs	Intravenous	10^6^ cells/mouse	Alleviated oxidative stress-induced mitochondrial dysfunction in the airways	[72]
Asthma	Mice model	iPSC-MSCs mesenchymoangioblast-MSCs	Intranasal	10^6^ cells/mouse	Provided greater protection against experimental chronic allergic airways disease compared with a clinically used corticosteroid	[73]
COPD	Mice model	Pioglitazone pretreated WJ-MSCs	Intravenous	10^4^ cells/mouse	Produced greater lung regeneration, compared to non-augmented WJ-MSCs, in a mouse emphysema model	[74]
COPD	Mice model	WJ-MSCs	Intravenous	5 × 10^4^ cells/mouse	They didn’t confirm the effects of WJ-MSCs in COPD through this experiment	[75]
COPD	Mice model	HCB-MSCs	Intravenous	5 × 10^4^ cells/mouse	Improved the regenerative mechanisms based on the gene expression profile changes	[76]
Asthma	Mice model	HCB-MSCs	Intravenous	10^5^ cells/mouse	Suppressed severe asthma by directly regulating Th2 cells and type 2 innate lymphoid cells	[77]
Asthma	Mice model	AD-MSCs BM-MSCs	Intravenous	2.5 × 10^7^ cells/Kg	Suppressed AHR and airway inflammation and induced eosinophilic airway inflammation and lung histological changes	[81]
Asthma	Mice model	AD-MSCs	Intratracheal	10^6^ cells/mouse	Alleviated airway inflammation, improved airway remodeling, and relieved AHR	[17]
Asthma	Mice model	AD-MSCs	Intravenous	10^5^ cells/mouse	Reduced lung inflammation and remodeling while causing immunosuppression	[82]
Asthma	Feline model	AD-MSCs	Intravenous	2 × 10^6^, 4 × 10^6^, 4.7 × 10^6^ and 10^7^ cells/cat	Had a delayed potential in decreasing airway inflammation, AHR and remodeling	[83]
Asthma	Mice model	HAM-MSC-CM	Intravenous	10^6^ cells/mouse	Reduced inflammatory factors and fibrosis	[84]
Asthma	Rat model	HP-MSCs	Intraperitoneal	10^6^ cells/Kg	Suppressed airway inflammation in asthmatic rats by modulating Notch signaling	[85]
Asthma	In vitro	HP-MSCs	–	–	Reduced the IL-5 level experimentally in children with asthma	[86]
Asthma	Rat model	HP-MSCs	Intravenous	1 × 10^7^ cells/ml	Improved AHR and inflammation by modulating the Th17/Treg balance	[87]
Asthma	In vitro	DF-MSCs	–	–	Down-regulated Th2-mediated immune response in asthmatic patients mononuclear cells	[88]
COPD	Mice model	BM-MSCs and BM-MSC-Exos	Intraperitoneal	10^6^ cells	Combination treatment may act against early events caused by CS exposure owing to its anti-inflammatory and other mitochondrial transfer mechanisms	[89]
Asthma	In vitro	BM-MSC-Exos	–	–	Promoted immunosuppression of regulatory T cells	[90]
Asthma	Rat model	BM-MSCs and BM-MSC-Exos	Intravenous	5 × 10^6^ cells/cat	Reduced airway remodeling in lungs through the Wnt/β-catenin signaling pathway	[91]
Asthma	Mice model	BM-MSC-Exo-miR-188	–	–	Reduced bronchial smooth muscle cell proliferation in asthma through suppressing the JARID2/Wnt/β-catenin axis	[92]
Asthma	In vitro	BM-MSC-Exo-miR-146a-5p	–	–	Inhibited Th2 differentiation via regulating miR-146a-5p/SERPINB2 pathway	[93]
Asthma	Mice model	AD-MSC-EVs	Intranasal	10 μg	Alleviated AHR and allergic airway inflammation caused by the induction of Treg expansion	[94]
Asthma	Mice model	AD-MSC-Exo-miR-301a-3p	–	–	Regulated airway smooth muscle cells by targeting STAT3	[95]
Asthma	Mice model	AD-MSC-EVs	Jugular	37 μg	Acted differentially on lung mechanics and inflammation in experimental allergic asthma	[96]
Asthma	Mice model	mmu_circ_0001359-modified AD-MSC-Exos	Intravenous	200 μg	Attenuated airway remodeling by enhancing FoxO1signaling-mediated M2-like macrophage activation	[97]
Asthma	Mice model	iPSC-MSC- EV-miR-146a-5p	Intravenous	100 µg	Prevented group 2 innate lymphoid cell-dominant allergic airway inflammation	[98]
Asthma	Mice model	Hypoxic-hUC-MSC-EVs	Intravenous	40 μg	Attenuated allergic airway inflammation and airway remodeling	[99]
Asthma	RAW 264.7 cell line	HUC-MSC-Exos	–	–	Attenuated the inflammation of severe steroid-resistant asthma by reshaping macrophage polarization	[100]
COPD	Mice model	P-MSC-Exo-MAPPS	–	–	Enhanced pulmonary function through decreasing serum concentrations of inflammatory cytokines, lung-infiltrated macrophages, neutrophils, and natural killer and antigen-presenting cells and elevated anti-inflammatory IL-10 and (Tregs)	[101]
Asthma	Mice model	hP-MSC-Exos	Intranasal	50 μg	Expanded lung IL-10-producing IMs, which may originate from spleen, thus contribute to protection against asthma	[102]

**Table 2:** Clinical application of various mesenchymal stem cells in COPD patients

Injury	Enrollment number	Design and phase of study	Type of MSCs	Following duration	Infusion method	Dose of injection	Outcome	NCT number	Reference
COPD	9	Matched-control	Autologous BM-MSCs	–	–	–	Feasible	NCT01306513	[56]
COPD	10	Phase I, prospective open-label	Autologous BM-MSCs	3 weeks	Intravenous	1–2 × 10^6^ cells/kg	Feasible and safe	NCT01306513	[57]
COPD	10	Phase I, prospective, nonrandomized, patient‐blinded, placebo‐controlled	Allogeneic BM-MCs	90 days	–	10^8^ cells/kg	Feasible and safe	NCT01872624	[58]
COPD	4	Phase I	Autologous BM-MCs	3 years	Intravenous	10^8^cells/kg	Feasible and safe	NCT01110252	[59]
COPD	9	Phase I pilot study	Allogeneic BM-MSCs	1 year	Intravenous	2 × 10^6^ cells/kg	Feasible and safe	12,614,000,731,695	[60]
COPD	62	Randomized, placebo	Allogeneic BM-MSCs	2 years	–	–	Feasible	NCT00683722	[62]
COPD	62	Placebo-controlled, randomized, double-blinded	Allogeneic BM-MSCs	2 years	Intravenous	10^8^ cells/kg	Feasible and safe	NCT00683722	[61]
COPD	40	Matched case–control, phase I/II trial	Allogeneic HUC-MSCs	1 year	Intravenous	10^6^ cells/kg	Feasible and safe	NCT04433104	[78]
COPD	20	Controlled, pilot clinical trial	Allogeneic HUC-MSCs	6 months	Intravenous	10^6^ cells/kg	Feasible and safe	ISRCTN70443938	[79]
COPD	20	Matched-control	AD-MSCs and autologous BM-MCs	–	Intravenous	10^8^ cells/kg	Feasible and safe	NCT02412332	[80]

### Bone marrow-MSCs

#### COPD

In 2016, an in vitro study provided evidence that conditioned media (CM) from BM-MSCs stimulated with pro-inflammatory cytokines such as TNF-α and IL-1β is a potential method to enhance the regeneration of airway epithelial wound in NCI-H292 cells and could be beneficial in COPD treatment which may mainly mediated by induction of hepatocyte growth factor (HGF) and epidermal growth factor receptor (EGFR) and subsequent activation of the ERK1/2 signaling pathway [48]. Recently, Hong-mei LIU et al. (2015) reported that BM-MSC administration could regenerate lung damage via promoting proliferation of endogenous lung stem cells in COPD mice induced by the CS [49]. Additionally, a recent finding has demonstrated the therapeutic impact of rat BM-MSCs intrapulmonary administrated into the lungs of CS-induced emphysematous rats by downregulation of the cell apoptosis, inflammation and protease expression as well as increase of vascular endothelial growth factor (VEGF) and transforming growth factor-β1 (TGF-1). Nonetheless, the authors declared that these MSCs did not fully reverse emphysema. Besides, single injection of BM-MSCs to rats was a drawback of this experiment [50]. In another animal study, BM-MSCs ameliorated lung damage in acute exacerbation COPD mice model by anti-bacterial effects and relieving inflammation reaction which may be mediated by secretion of TSG-6, inhibition of NF-кB signaling and enhancement of macrophages phagocytosis via paracrine mechanisms [51]. It has also been reported that intrabronchial BM-MSC transplantation relived airway inflammation and emphysema in CS-exposed rat models via regulation of cyclooxygenase-2 (COX-2) and PGE2 synthesis in alveolar macrophages by p38 and ERK MAPK signaling pathways. However, the authors did not elucidate the role of JNK in reduction of COX-2 [52]. Kennelly et al. [53] also revealed that human BM-MSC therapy could protect against COPD and decrease lung injury by significant anti-inflammatory, anti-fibrotic and anti-apoptotic functions which are associated to elevation of hepatocyte growth factor (HGF) in mouse model of COPD. Chen et al. [54] established that intravenous injection of BM-MSCs could alleviate emphysema through differentiating into endotheliocytes, reducing their apoptosis and oxidative stress in rats with overlap syndrome after four weeks. Nonetheless, it is unclear that if multiple administration of undifferentiated BM-MSCs elevate risk of cancer. It has also been indicated that cis-resveratrol (c-RSV)-treated VEGF factor A (VEGFA) expression in heat-shock protein (HSP)-VEGFA- transduced BM-MSCs considerably enhanced the therapeutic impacts in mouse with COPD through downregulation of inflammatory cytokines, elevation of anti-oxidant genes and regeneration of lung cells [55].

Several clinical trials have also been studied the role of BM-MSCs in COPD patients. For instance, in a study conducted by Broekman et al. [56], the therapeutic potential of autologous BM-MSCs from COPD patients were evaluated. According to the results, these MSCs were phenotypically and functionally comparable to those MSCs from healthy controls and may be suitable choices for COPD treatment. However, they found several differences including IL-6 secretion, adipocyte differentiation and NQO1 expression via Nrf2-ARE pathway. A phase I, prospective open-label trial (NCT01306513) in Netherlands assessed the safety and feasibility of intravenous infusion of two doses of autologous BM-MSCs (1–2 × 10^6^ cells/kg) after and prior to one-sided lung volume reduction surgery (LVRS) and a second LVRS procedure, respectively in 10 subjects with severe lung emphysema. All participants showed elevated FEV1 and no adverse effects and fibrotic reactions were observed after one-year follow-up. Nevertheless, lack of a placebo group was the major drawback of this study. Moreover, since endothelial injury is a prominent sign of emphysema, they used just CD31 as endothelial cells marker, however, it is not limited to these cells alone and extra markers are required to investigate this result. Additionally, the trial was not randomized and placebo-controlled and a larger number of patients were needed [57]. Oliveira et al. (2016) conducted a placebo-controlled, nonrandomized phase I clinical study involving 10 patients with severe COPD and aged between 40 to 80 years who recruited from Hospital de Clinicas de Porto Alegre, Brazil (ClinicalTrials.gov identifier: NCT01872624). Five participants bronchoscopically received combined BM-MSC (10^8^ cells) therapy with one-way endobronchial valves (EBV) insertion and the remaining 5 patients received saline as the control and monitored up to three months. The results of this study indicated that BM‐MSC transplantation appeared to be safe and made no remarkable change in frequency of COPD exacerbations, severity of disease and radiological outcomes. Nonetheless, the C-reactive protein (CRP) levels was decreased in days 30 and 90 and quality of life was improved in the BM‐MSC-treated group. Short following-up period, low number of participants, and evaluation of inflammatory mediators only in plasma was the major disadvantages of this study [58]. Similarly, treatment of COPD patients with BM-derived mononuclear cells (BM-MCs), heterogeneous pool of cells which contains MSCs, has displayed emerging outcomes. In 2013, one phase I clinical trial has been performed by Universidade Estadual Paulista (UNESP) in Assis, SP, Brazil (ClinicalTrials.gov, NCT01110252). In this study, collected autologous BM-MCs (10^8^ cells/kg) were intravenously injected to four patients with advanced COPD and followed up for 3 years. No adverse effects were found in these patients and half of them showed an improvement in FEV1. Despite the small sample, these enhancements in pulmonary function could be as result of anti-inflammatory properties of BM-MC therapy [59]. A phase I pilot study has also been designed to determine the allogenic BM-MSCs effect in treatment of 9 patients with moderate to severe COPD (Australian clinical trials registry no. 12614000731695). BM-MSCs (2 × 10^6^ cells/kg) was intravenously infused to the participants twice times and followed up to 12 months. This therapy led to alleviation of COPD via immunomodulatory effects and decrease of inflammation by releasing EVs and no adverse effects were detected [60]. One placebo-controlled, randomized clinical trial investigated the safety and the effect of allogeneic BM-MSCs for severe COPD therapy in 62 individuals between 40 to 80 years enrolled from different institutions in the United States; http://clinicaltrials.gov, NCT00683722. The participants received four monthly administrations (10^8^ cells/per infusion) and no deaths or serious adverse events were found during the 2‐year follow‐up period. Besides, the serum levels of circulating CRP was significantly reduced in patients received MSCs therapy at study entry. Nevertheless, no considerable enhancement was observed in COPD patients who received MSC treatment [61]. Moreover, a randomized clinical article by these authors have also published in 2021 to evaluate the impact of allogenic BM-MSC transplantation in 62 COPD patients with high levels of CRP (NCT00683722). In contrast with the results of previous study, they showed significant efficiency of BM-MSCs in pulmonary function improvements [62].

#### Asthma

An experimental study by Mohammadian et al. in 2018 demonstrated that a single infusion of simvastatin in combination with BM-MSCs could remarkably reduce serum total and specific IgE, as well as lung IL-13 and TGF-β levels in ovalbumin-induced murine models of asthma as compared with simvastatin alone. The excellent effects of combination therapy may be because of elevation of BM-MSC migration to lung [63]. In another work, immunoregulatory effects of BM-MSCs and mitochondrial signaling pathways was investigated in asthmatic mice model through both intratracheal and systemic routs. The authors proposed that BM-MSC therapy is a potential strategy in preventing lung damage through suppressing the eosinophils, inflammatory cytokines, mucus hyper-production and the mitochondria genes expression, but increasing IFN-γ. However, there were some drawbacks including not studying the BM-MSCs on chronic lung inflammation and asthma and also remodeling associated biofactors were not done [64]. Abreu and coworkers (2017) compared the therapeutic efficiency of intratracheal injection of MSCs derived from BM, AD, and lung in ovalbumin-induced mice model of asthma. Their result showed that BM-MSCs are more potent in decrease of inflammatory cytokines, eosinophils, and VEGF in lung in comparison with AD- and lung-MSCs. Furthermore, only BM-MSCs were able to increase IL-10 and IFN-γ in lung. Results of in vitro study also showed that BM-MSCs augmented polarization of macrophages toward M2. More researches are needed for evaluation of various MSCs potential in phagocytosing macrophages differentially. Whereas, MSCs from AD and lung had higher baseline levels of IL-4, insulin-like growth factor (IGF), and VEGF secretion [65]. It has also been evidenced that intratracheal administration of rat BM-MSCs and conditioned media (CM) have potential role in improving pathological changes in ovalbumin-sensitized asthmatic rats probably through modulating ICAM-1 and VCAM-1 expression in lung tissues. Results also represented that rat BM-MSCs pose more regenerative abilities than that of CM. However, there were several restrictions regarding this study such as not investigation of ICAM-1 and VCAM-1 expression on circulating leukocytes and not measuring of protein levels of both molecules and signaling pathways at the downstream. Furthermore, the content of CM was not examined completely and it is better to address the presence of modulating factors in the future studies [66]. Another research conducted by Işık et al. (2016) proved that BM-MSC administration via intraperitoneal route could ameliorate ovalbumin-induced allergic rhinitis in the murine model through migrating to lung and nasal tissues. They proposed that the BM-MSCs enhanced to the airway remodeling and inflammation through the downregulation of Th2 cells [67]. It has also been indicated that prevention of Notch1/Jagged1 pathway improve BM-MSC homing to enhance asthma in rats through regulating Th1/Th2 drift [68]. The therapeutic effect of BM-MSCs have also been assessed in ovalbumin-induced allergic asthma and cytokine responses in mice. The authors found that the BM-MSCs could considerably decrease the total cells, eosinophilia and serum IgE secretion by inhibiting Th2 and Th17 cytokines and elevating of Treg cytokines concentration [69]. In addition, BM-MSCs have shown immunosuppressive effects on asthma, that is mediated by TGF-β-signaling-dependent alveolar macrophage polarization [70]. Furthermore, it was found by Goldstein et al. that human BM-MSCs are able to decrease chronic inflammation in an ovalbumin-induced murine asthma model through decreasing extracellular matrix (ECM) deposition as evidenced by reductions in soluble and insoluble collagen synthesis [71].

### Induced-pluripotent stem cell-MSCs

#### COPD

In a report by Li et al. [72], iPSC-MSCs were intravenously administrated into the mice with pulmonary obstruction exposed to ozone. According to the results, a remarkable reduction was observed in oxidative stress-induced mitochondrial dysfunction as well as decreased in airway inflammation and airway hyper-responsiveness (AHR) in the murine lungs. Moreover, in vitro results showed that human airway smooth muscle cells (ASMCs)-treated iPSC-MSCs significantly downregulate mitochondrial reactive oxygen species (ROS) and apoptosis in these cells. Nevertheless, the in vitro and in vivo models did not indicate the condition in COPD lungs. Moreover, in the lack of CS, iPSC-MSCs augmented ASMCs apoptosis probably by inducing stress in these cells at baseline, implying that the safety of the MSCs must be carefully analyzed.

#### Asthma

A recent finding has demonstrated that intranasal administration of iPSC- and mesenchymoangioblast-derived MSCs provided superior protection against ovalbumin-induced chronic allergic airways disease/asthma than that of clinically used corticosteroid [73].

### Wharton’s jelly-MSCs

#### COPD

Park et al. (2018) evaluated the therapeutic efficiency of WJ-MSCs in treatment of two mouse emphysema models, an elastase-induced and a CS-induced models [74]. The authors demonstrated that intravenous administration of pioglitazone pretreated WJ-MSCs (10^4^ cells) led to better pulmonary regeneration as compared with non-treated cells. In a similar study, Cho and coworkers [75] have investigated the efficiency of WJ-MSC therapy for lung recovery in COPD murine models. The mice were divided into three groups including sham group that was not induced COPD, nor received any therapy, COPD mice received saline as controls, and COPD mice received WJ-MSCs (5 × 10^4^ cells) intravenously. Authors declared that no significant effects of WJ-MSC therapy was found in this experiment that may be related to the low number of animals. Another drawback of this study was that no mechanism underlying the effect of WJ-MSCs was not identified.

### Human cord blood-MSCs

#### COPD

In a study conducted by You-Sun Kim et al. [76], systemic injection of hCB-MSCs into a CS-induced COPD mouse model, were shown to have regenerative effects based on the gene expression profile changes. According to the results of this study, immune responses, oxidative stress, and transcription were regulated in the lung cells on days 1 and 4 after hCB-MSC administration, whereas blood vessel growth were regulated at a later stage (on day 14) in comparison with controls. However, there is a lack of mice group exposed to normal air to exact identification of regeneration mechanisms of hCB-MSCs. In addition, their microarray experiment utilized whole lungs and therefore, gene profiles change in individual cells remained uncertain.

#### Asthma

Shin et al. assessed the beneficial effects of hUC-MSC therapy in two murine models of severe asthma, which ameliorated via suppressing inflammation through Th2 lymphocytes. Moreover, in vitro results indicated that hUC-MSCs directly reduced the IL-5 and IL-13 levels of differentiated mouse Th2 lymphocytes and peripheral blood mononuclear cells (PBMC) from asthmatic patients [77].

### Umbilical cord-MSCs

#### COPD

Hoang et al. (2021) performed a matched case–control phase I/II trial involving 40 patients with mild-to-severe COPD at Vinmec Times City International Hospital, Hanoi, Vietnam (NCT04433104). Twenty patients were intravenously administrated twice by UC-MSCs as intervention therapy with an interval of three months and at a dose of 10^6^ cells/kg. Twenty other participants were also recruited as control group and followed up for one year. This study provided data supporting that UC-MSC therapy is a safe and efficient treatment for COPD patients. However, it was not conducted as a randomized trial which is a drawback of this study [78]. Likewise, Bich and coworkers published a pilot clinical study registered in ISRCTN with a registration number of ISRCTN70443938 which explored the safety and efficiency of allogeneic UC-MSCs in 20 patients suffering from mild-to-severe COPD [79]. A single dose of UC-MSCs (10^6^ cells/kg) were injected to the participants and followed for 6 months. No severe adverse effects or mortality were happened that were deemed correlated with UC-MSC infusion. Moreover, the treated patients displayed a considerably decreased Modified Medical Research Council score, COPD assessment test, and number of exacerbations. Nevertheless, CRP and the FEV in 1 s (FEV1) were not showed any significant reduction in these patients after therapy in comparison with those before the treatment.

### Adipose tissue-MSCs

#### COPD

A more recent publication in 2021 confirmed the therapeutic potential of MSC therapy in COPD patients (NCT02412332). In this randomized, open-controlled phase I clinical trial, 20 participants suffering from grade 3 COPD were recruited from pulmonology outpatient clinic of Faculdade de Medicina ABC (ABC Medical School, Brazil) and Instituto Chico Anysio (Rio de Janeiro, Brazil) and divided to four groups: 1. five individuals received conventional therapy, 2. five individuals received BM-MCs, 3. five individuals received adipose-derived (AD)-MSCs, and 4. five individuals received the co-administration of BM-MCs and AD-MSCs. No side effects linked to MSC therapy was detected after one-year follow-up. The BM-MC-derived patients indicated elevated FEV1 and diffusing capacity for carbon monoxide (DLCO). Besides, the co-administrated group demonstrated DLCO and better life quality [80].

#### Asthma

Results of an experimental research indicated that AD-MSCs and BM-MSCs could suppress AHR and airway inflammation in ovalbumin-induced mice. Nevertheless, double MSC therapy considerably induced eosinophilic airway inflammation and lung histological alterations and then, it is not effective against asthma [81]. The beneficial effects of AD-MSCs have also evaluated in ovalbumin-sensitized asthmatic murine model. Intratracheal injection of these MSCs relieved airway inflammation, AHR and enhanced airway remodeling which may be related to the regulation of Th1/Th2 cell balance [17]. Since dust mite or Alternaria animal models display a closer similarity to human asthma, it’s better to analyze the therapeutic effects of MSCs in these models in studies. For example, Castro et al. (2020) have highlighted the therapeutic impacts of multiple-dose human AD-MSCs injected systemically in an experimental model of house dust mite‐induced allergic asthma which attenuated pulmonary inflammation and remodeling but contributing to immunosuppression [82] In 2016, a pilot study by Trzil et al. was also conducted to investigate the feasibility and efficacy of five intravenous administrations of AD-MSCs in an experimental feline asthma model. They proposed that the AD-MSCs could have a delayed potential in decreasing airway inflammation, AHR and remodeling [83].

### Human amniotic membrane-MSCs

#### Asthma

As shown by Dalouchi et al. (2021) in their article, hAM-MSC-CM notably modulated fibrosis, oxidative stress, and inflammation via decreasing the level of eosinophils and neutrophils, IL-4, and TGF-β in ovalbumin-induced asthma murine model. The results also indicated that the MSC-CM elevated the IFN-γ and IL-10 [84].

### Human placental-MSCs

#### Asthma

With an ovalbumin-induced rat model of acute asthma, injection of hP-MSCs exerted beneficial effects on asthmatic rats and suppressed inflammation by modulating Notch signaling pathway [85]. Besides, the results of this study showed that serum IFN‑γ notably elevated after hP-MSCs transplantation, whereas IL-4 and IgE reduced. Another in vitro study indicated that hP-MSCs could attenuate the proliferation and activation of CD4^+^ and CD8^+^ T cells and decreased IL-5 level in culture in different subgroups of children with asthma. However, this study was performed on small number of children and only analyzed IL-5 [86]. Another animal study by Li and coworkers [87] revealed that Th17/Treg balance were regulated following hP-MSC administration and improved AHR and inflammation in ovalbumin-induced asthmatic rats.

### Dental follicle-MSCs

#### Asthma

In an in vitro study by Genç et al. (2018), the immunoregulatory potential of DF-MSCs was investigated through isolating PBMCs from healthy and asthmatic patients. They found that the DF-MSCs suppressed Th2 mediated immune responses and IL-4 cytokine and elevated Tregs and IFN- γ level [88]. Furthermore, it was indicated that the DF-MSCs decreased inflammation by IDO and TGF-β pathways in asthmatic patients.

## Various MSC-EVs in treatment of COPD and asthma

Table 1 also summarized the studies employed various MSC-EVs in treatment of COPD and asthma disorders which are discussed in the following parts.

### Bone marrow-MSC-EVs

#### COPD

The therapeutic effects of intraperitoneal co-administration of BM-MSCs and BM-MSC-Exos was examined by Maremanda et al. [89] in CS-induced mitochondrial dysfunction in COPD mouse. According to the findings of this study, the combination therapy alleviated COPD through anti-inflammatory and targeting mitochondrial genes and indicated protection in comparison with BM-MSCs or their exosomes alone.

#### Asthma

In a research, the immunomodulatory impacts of BM-MSC-Exos evaluated on PBMCs of patients suffering from asthma. Obtained data elucidated that the MSC-Exos increase IL-10 and TGF-β1 from PBMCs, therefore improving proliferation and immunomodulation capability of Treg cells [90]. In addition, Song and coworkers showed that administration of BM-MSCs and BM-MSC-Exos into the ovalbumin-induced asthma rat model could notably prevent chronic airway inflammation, decrease remodeling and EMT in the lung epithelial cells via suppressing Wnt/β-catenin signaling pathway [91]. It has also been recently shown that exosomal miR-188 from BM-MSC-derived exosomes could attenuate proliferation of bronchial smooth muscle cells and pulmonary damage in asthmatic ovalbumin-induced murine models by the JARID2/Wnt/β-catenin axis. Besides, the exosomes considerably decreased the abnormal proliferation and migration of TGF-β1-tread bronchial smooth muscle cells in vitro [92]. Zhou et al. (2021), also found that exosomal miR-146a-5p released from BM-MSCs could prevent the Th2 cells differentiation by regulating the SERPINB2 pathway in the blood sample of allergic rhinitis patients [93].

### Adipose tissue-MSC-EVs

#### Asthma

A recent research from Mun et al. [94] discovered that intranasal injection of AD-MSC-EVs have immunoregulatory impacts in a murine model of ovalbumin-induced asthma via attenuating AHR and inflammatory responses caused by the induction of Tregs. However, they could not differentiate if these effects were associated with exosomes, microvesicles, or a combination of these. In an experiment by Feng et al. [95], the therapeutic potential of AD-MSC-Exo-derived miR-301a-3p was investigated in ovalbumin-induced asthma murine model. They reported that the exosomes were efficiently internalized by airway smooth muscle cells and their secreted miR-301a-3p significantly down-regulated the inflammation, platelet-derived growth factor-BB (PDGF-BB), stimulated proliferation and migration and increased apoptosis in these cells through targeting STAT3. Furthermore, systemic administration of AD-MSC-EVs are associated with reduction of fibrosis and eosinophil counts in lung tissue and airway remodeling, however, their impacts on T cells differed in lung and thymus of allergic asthma ovalbumin mouse model. Nonetheless, AD-MSCs or EVs were not tracked following injection and therefore limited knowledge concerning their delivery and homing. Besides, the efficiency of only a single dose was investigated that is another limitation of this study [96]. Another animal study showed that exosomes from mmu_circ_0001359-modified AD-MSCs could significantly attenuate airway remodeling by improving FoxO1 signaling-mediated M2-like macrophage activation, by sponging miR-183-5p [97].

### Induced-pluripotent stem cell-MSC-EVs

#### Asthma

Fang and coworkers [98] declared that small EV-miR-146a-5p from human iPSC-MSCs could inhibit group 2 innate lymphoid cell (ILC2)-dominant allergic airway inflammation in mouse model and in vitro.

### Human umbilical cord-MSC-EVs

#### Asthma

Dong et al. administrated EVs released from Nor- and Hypoxic-hUC-MSCs to two groups of ovalbumin-induced mice models of chronic allergic asthma. The control group was also received PBS. They found that Hypo-EV-treated group were more significantly potent in suppression of the pro-inflammatory cytokines such as IL-4 and IL-13, and eosinophils and attenuating airway remodeling via decreasing expression of pro-fibrogenic markers than that of Nor-EVs. Additionally, it was demonstrated that miR-146a-5p enriched in Hypo-EVs could mediate lung protection in ovalbumin mice. These alternations may contribute to enhanced outcomes for hUC-MSC-EVs related to inhibition of TGF-β1/Smad2/3 signaling pathway and protection of airway inflammation and fibrosis [99]. It has been demonstrated that intratracheal infusion of hUC-MSC-Exos into severe steroid-resistant asthma (SSRA) mice suppress airway inflammation and AHR. In vitro results of this study also reported that these exosomes could upregulate M1 polarization to M2 in LPS-stimulated RAW 264.7 cells and the underlying mechanism may be modulating the activation of NF-κB and PI3K/AKT signaling through targeting TRAF1 [100].

### Placental-MSC-EVs

#### COPD

For example, in an animal study conducted by Harrell et al., molecular and cellular mechanisms of P-MSC-Exo-derived multiple allogeneic protein paracrine signaling (MAPPS) was evaluated in CS-exposed mice models of COPD. The P-MSC-Exo-MAPPS considerably enhanced pulmonary function through decreasing serum concentrations of inflammatory cytokines (TNF-α, IL-1β, IL-12, and IFN-γ), lung-infiltrated macrophages, neutrophils and their secreted serine proteases, and natural killer and antigen-presenting cells. In addition, these products elevated anti-inflammatory IL-10 and regulatory T cells (Tregs) in COPD mice. The therapeutic potential of P-MSC-Exo-MAPPS was further investigated in 30 COPD patients and no adverse effects were found. The results also showed enhanced lung status and quality of life in these individuals [101].

#### Asthma

Ren et al. [102] elucidated that twice intranasal delivery of hP-MSC-Exos could be employ as emerging therapeutic tools for the treatment of an ovalbumin-induced murine model of asthma via their immunomodulatory effects which were due to the expansion of IL-10 producing lung interstitial macrophages.

## Conclusion and future perspective

In recent years, MSCs have gained prominent implications in treatment of different respiratory diseases with regard to their therapeutic abilities such as anti-inflammatory and immunoregulatory properties, regenerative capacity as well as many other beneficial features. Accumulating researches have been assessed the safety and efficiency of MSCs from different sources such as BM, adipose tissue, hUC, UCB, WJ, placental, etc., in COPD. Although, more clinical studies are still needed to warrant the safety and feasibility of MSC therapy especially in asthma prior to their routine application in the clinic. Small number of recruited participants was also a prominent limitation in most of the clinical trials.

BM-MSCs were the most prevalent source employed for assessments in lung disorders. In addition, because of their lower tumorigenicity, immunogenicity, and easier management, EVs, especially exosomes, may be a safer and effective therapeutic tools for treatment of lung conditions as compared with their parental MSCs. Nevertheless, the underlying mechanism in MSC-EVs biogenesis, pharmacokinetics and biodistribution requires very extensive investigates prior to the application of this approach in clinic. Further comprehensive experiments are also required to clarify the optimal protocol for isolation and preparation of MSCs for clinical use. Furthermore, modification or engineering of MSC-derived EVs can make them emerging therapeutic candidates for decreasing undesired adverse events in the future clinical use of MSC‐EVs.

## Data Availability

Not applicable.

## Abbreviations

AHR: Airway hyper-responsiveness
ASMCs: Airway smooth muscle cells
AD: Adipose tissue
BM: Bone marrow
BM-MCs: Bone marrow-derived mononuclear cells
COPD: Chronic obstructive pulmonary disease
c-RSV: Cis-resveratrol
CRP: C-reactive protein
CM: Conditioned media
CS: Cigarette smoke
DLCO: Diffusing capacity for carbon monoxide
EVs: Extracellular vesicles
ECM: Extracellular matrix
EBV: Endobronchial valves
FEV: Forced expiratory volume
HGF: Hepatocyte growth factor
Hi-PSC: Induced pluripotent stem cells
hUC: Human cord blood
hAM: Human amniotic membrane
hP: Human placental
HSP: Heat-shock protein
IGF: Insulin-like growth factor
LVRS: Volume reduction surgery
MSCs: Mesenchymal stem cells
MVs: Microvesicles
MPs: Microparticles,
MAPPS: Multiple allogeneic protein paracrine signaling
PBMC: Peripheral blood mononuclear cells
PDGF-BB: Platelet-derived growth factor-BB
ROS: Reactive oxygen species
SSRA: Severe steroid-resistant asthma
TGF-1: Transforming growth factor-β1
UCB: Umbilical cord
VEGF: Vascular endothelial growth factor
VEGFA: Vascular endothelial growth factor A
WHO: World health organization
WJ: Wharton’s Jelly
